# Dr. Crouse on Expenses to the Congress

**Published:** 1893-08

**Authors:** 


### DR. CROUSE ON EXPENSES TO THE CONGRESS.

We desire to call special attention to Dr. Crouse's communication under the head of "Domestic Correspondence," giving the facts regarding expenses at Chicago. There has been a mistaken impression abroad regarding this, and it is well to have the matter set right by so good an authority.

The few days which will elapse before the World's Columbian Congress will meet should be days of active preparation by every dentist to make one in that great body. It is presumed that before this reaches our readers the railroads will have reduced the fare in every direction. If this be done with some regard for the comfort of travellers there should be a large delegation from the East.

We have it from excellent authority, outside of Dr. Crouse, that the expense attending the convention need not be greater than that of the ordinary annual convocation.

If this Congress will have the effect of educating dentists to the advantages to be desired from attending annual gatherings, it will have performed an educational service not considered primarily as one of its good results. The apathy felt in dental circles regarding their annual meeting, both North and South, is not altogether creditable to a profession calling itself scientific. There is, therefore, much to hope from the training and experience which this week at Chicago will give to those not familiar with the work, and we hope not hundreds but thousands will avail themselves of the opportunity.

